# Supplementary material for: Controlled Trial Examining the Strength-Based Grit Wellbeing and Self-Regulation Program for Young People in Residential Settings for Substance Use
Source: Int J Environ Res Public Health. 2022 Oct 24;19(21):13835. doi: 10.3390/ijerph192113835 (PMC9657698; doi:10.3390/ijerph192113835)
Supplement: Supplementary file 1 [file ijerph-19-13835-s001.zip › ijerph-1928728-supplementary.pdf]

Table S1. Session Details for Grit Wellbeing and Self-regulation Program

Session Number and Name		Wellbeing Target	Content	Reference
1	Learning to Breath-Ground-Centre	Emotional; Psychological; Physical	Clients learn practical methods to ground themselves and remain in the present moment. The sessions has a particular emphasis on breathing techniques, grounding methods, and ways to identify their centre or core.	Rock and Water program (Raymond, 2005; Ykema, 2002)
2	Focus/Mind Fit	Psychological; Physical	Clients learn the final part of "being present", which is focussing. They also experience up to four practical mindfulness tasks (i.e., a guided meditation, mindful boxing, mindful eating, mindful walking).	Rock and Water program (Raymond, 2005; Ykema, 2002) mindfulness-based relapse prevention programs (Bowen, Chawla, & Marlatt, 2011; Roos et al., 2019).
3	Social Identity and Social Support	Social	Clients learn the importance of social support and complete an exercise to map out their social world (social identity mapping task), including identifying importance, positivity, level of support, representativeness, and compatibility of their social connections.	Groups for Health program (Haslam et al., 2019; Haslam, Cruwys, Haslam, Dingle, & Chang, 2016), and used in other residential AOD treatment settings (Best et al., 2016; Dingle et al., 2020; Mawson et al., 2015).
4	Character Strengths	Social; Psychological	This session begins with group exercises to draw out character strengths. Clients then learn how to identify their own strengths and the strengths of others and identify what strengths they would like to build and develop.	Strengths-based mindfulness approach (Niemiec, 2012)
5	Social Wellbeing	Social	Clients identify barriers to group belonging and connecting, and areas of compatibility/incompatibility and loss in their social networks. Clients also learn the importance of giving and receiving support in relationships.	Groups for Health program (Haslam et al., 2019; Haslam, et al., 2016), Social Identity Mapping (Best et al., 2016; Dingle et al., 2020; Mawson et al., 2015).
6	Identity and Values	Psychological	Clients identify important parts of their self-concept and examine how their social connections and strengths represent those aspects of themselves. Clients also engage in an exercise identifying their values and identify how much they are living in alignment with their values.	ACT Approach - (Hayes, Strosahl, & Wilson, 1999; Harris, 2013)
7	Communication	Social; Psychological	Clients learn about the different communication styles (passive, aggressive, and assertive); strategies for effective communication; how to be assertive; and, tips on having difficult conversations. Clients also examine their own and others' boundaries, and ways to establish boundaries.	
8	Emotions and Music	Emotional	Clients learn the connection between music and emotion, using music to identify a range of emotions. Clients also learn how to mindfully experience emotions and strategies to enhance joy and to savour positive experiences.	Tuned In music emotion regulation program (Dingle & Carter, 2017; Dingle & Fay, 2017; Dingle, Hodges, & Kunde, 2016); Music app (Music eScape; Hides et al., 2019).
9	Emotional Wellbeing	Emotional	Clients learn factors that underlie and impact their experience of emotion, understand the impact of substance use on emotions, and learn how to improve and manage unpleasant emotions by mapping mood and exercise journeys.	Tuned In music emotion regulation program (Dingle & Carter, 2017; Dingle & Fay, 2017; Dingle, Hodges, & Kunde, 2016); Music app (Music eScape; Hides et al., 2019).
10	Managing Thoughts and Cravings	Psychological; Physical	The session discusses the connection between thoughts, emotions, physical sensations, and behaviours. Clients learn strategies to manage unhelpful thoughts and cravings using mindfulness and breathe-ground-centre-focus exercises.	Mindfulness-based relapse prevention programs (Bowen, et al., 2011; Roos et al., 2019);
11	Healthy Me	Emotional; Psychological; Physical	This session revises the breathe-ground-centre-focus techniques, before focussing on trivia about physical activity, sleep and diet; and in particular,	

			how these are impacted by substance use and the importance of these health behaviours for wellbeing	
12	Difficult moments and developing Goals	Emotional; Social; Psychological; Physical	Clients learn practical strategies to aid them in managing conflict and dealing with difficult situations. They will also draw upon key components learnt throughout the program to develop goals through their recovery journey.	Mindfulness-based relapse prevention programs (Bowen, et al., 2011; Roos et al., 2019); Straight ahead: Transition skills for recovery. A Training Manual from the TCU/DATAR Project (Bartholomew, Simpson, & Chatham, 1993)
